# Prevalence of Depression, Anxiety, Stress, and Suicidal Ideation Among Pharmacy Students: An Updated Systematic Review and Meta-Analysis

**DOI:** 10.3390/ijerph23020155

**Published:** 2026-01-26

**Authors:** Titawadee Pradubkham, Julalak Klangpraphan, Patcharaporn Tangtrakuladul, Chatmanee Taengthonglang, Kritsanee Saramunee, Wiraphol Phimarn

**Affiliations:** 1 Clinical Pharmacy Research Unit, Faculty of Pharmacy, Mahasarakham University, Kantarawichai 44150, Thailand; titawadee.p@msu.ac.th; 2 Social Pharmacy Research Unit, Faculty of Pharmacy, Mahasarakham University, Kantarawichai 44150, Thailand; 63010710085@msu.ac.th (J.K.); 63010710046@msu.ac.th (P.T.); kritsanee.s@msu.ac.th (K.S.); 3 Pharmacy Department, Surin Hospital, Surin 32000, Thailand; chatmanee24@gmail.com

**Keywords:** pharmacy students, depression, anxiety, suicidal ideation, stress, systematic review, meta-analysis

## Abstract

**Highlights:**

**Public health relevance—How does this work relate to a public health issue?**

- Pharmacy students worldwide experience high levels of depression, anxiety, stress, and suicidal ideation, representing a substantial and underrecognized public health burden in this essential healthcare workforce.
- Significant regional and socioeconomic disparities show higher mental-health prevalence in low- and middle-income countries and resource-limited regions, underscoring inequities in mental-health vulnerability among future pharmacists.

**Public health significance—Why is this work of significance to public health?**

- Identifying mental-health problems in pharmacy students is crucial, as psychological distress can impair academic performance, professional competency, and the quality of pharmaceutical care.
- This study provides high-quality pooled evidence from 51 studies across 16 countries, offering a global perspective to guide policy, resource allocation, and strategies to strengthen the mental well-being of future healthcare providers.

**Public health implications—Key implications or messages for practitioners, policymakers, and researchers**

- Institutions and policymakers should prioritize evidence-based mental-health support systems such as screening, counseling, stress management, and academic support tailored to pharmacy students.
- Health authorities should address structural determinants such as economic disparities, academic pressures, and limited mental-health infrastructure and invest in longitudinal studies to guide targeted interventions for reducing psychological distress in this population.

**Abstract:**

Mental health conditions have become an increasing concern among university students, particularly those pursuing health science disciplines such as pharmacy. Rigorous academic demands, high workloads, and sustained psychological pressure place pharmacy students at a high risk of mental health disorders, including depression, anxiety, stress, and suicidal ideation. This study aimed to systematically review and quantitatively synthesize existing evidence on the prevalence of mental health conditions among pharmacy students in Thailand and globally using a meta-analytic approach. A comprehensive literature search was conducted across the major academic databases, including PubMed, ScienceDirect, Scopus, and ThaiJo, using predefined search terms and stringent inclusion criteria to ensure methodological rigor and relevance. Data from eligible studies were extracted and analyzed using STATA software to ensure statistical precision and reliability of the pooled estimates. A total of 51 studies, comprising 17,717 pharmacy students across 16 countries, including the United States, Thailand, Brazil, Malaysia, Syria, Pakistan, Poland, France, Portugal, Nigeria, Saudi Arabia, Sudan, Lebanon, Egypt, the United Arab Emirates, and Vietnam, were included. The meta-analysis revealed pooled prevalence rates of 44.26% for depression (95% CI: 36.08–52.61), 52.01% for anxiety (95% CI: 42.86–61.09), 48.10% for stress (95% CI: 32.96–63.43), and 24.52% for suicidal ideation (95% CI: 14.10–36.70). These findings reflect a substantial mental health burden among pharmacy students, necessitating immediate and context-specific interventions. Considering these findings, academic institutions must develop and implement comprehensive mental health support strategies. Such initiatives should include early identification and screening programs, access to psychological counseling services, resilience-building interventions, and stress management workshops to effectively address the psychological needs of pharmacy students and enhance their academic and personal well-being.

## 1. Introduction

Mental health issues are characterized by alterations in emotional regulation, cognitive function, and behavioral patterns that are often associated with psychological distress and impaired daily functioning or occupational performance. These conditions may also include suicidal ideation or self-harming behaviors [1]. The etiology of mental health disorders is multifactorial and encompasses a wide array of personal, social, and structural determinants. Personal factors include individual capacities for emotional regulation and genetic predispositions, while social and structural determinants include exposure to adverse environments, sociopolitical instability, economic inequality, and insufficient access to supportive communities or healthcare infrastructure. Collectively, these determinants may compromise mental well-being and increase the risk of mental health disorders [2].

According to the World Health Organization (WHO), approximately 970 million individuals were affected by mental health conditions globally in 2019. Notably, the prevalence of anxiety and depressive disorders surged by 26% and 28%, respectively, within a single year, underscoring the growing public health burden associated with these conditions [1]. A growing body of literature has highlighted the substantial mental health burden faced by pharmacy students, particularly depression and suicidal ideation. According to a recent study published in the American Journal of Pharmaceutical Education, a significant proportion of pharmacy students report experiencing depressive symptoms, with 13.5% claiming to experience suicidal ideation [3]. However, estimates of the prevalence of depression among this population vary widely across studies, ranging from 4.8% to 78.8%, reflecting considerable heterogeneity in study design, assessment tools, and contextual factors [4].

In addition, findings regarding the association between depressive symptoms or suicidality and demographic variables, such as academic year, sex, and other individual characteristics, remain inconsistent across studies, warranting further investigation. Accurate and reliable estimates of the prevalence of depression and suicidal ideation among pharmacy students are essential to develop targeted prevention, early detection, and intervention strategies. In a previous national study conducted in the United States, 35.1% of pharmacy students reported experiencing moderate to severe depressive symptoms, whereas 17.3% acknowledged experiencing suicidal ideation [3]. Furthermore, additional mental health concerns were observed, including clinically significant anxiety in 21% of pharmacy students in the same country [5]. The reported levels of stress were also notable, with 3.0% of the students categorized as experiencing intermediate-level stress and 2.8% as experiencing high-level stress [6].

Growing evidence indicates that mental health problems among pharmacy students are significantly associated with impaired academic performance and reduced productivity [7,8]. Pharmacy students may be particularly vulnerable to mental health difficulties due to the demanding nature of their training. Compared with other health science programs, pharmacy curricula often involve an intensive workload, extensive pharmacotherapy content, laboratory-based practical components, and high-stakes assessments, alongside clinical placement requirements, all of which contribute to elevated academic pressure.

Mental health disorders, particularly depression, have been shown to negatively affect cognitive functioning, concentration, and academic engagement, all of which are critical for the success of rigorous professional programs such as pharmacy. Psychological distress is associated with an increased risk of academic attrition. Students experiencing mental health challenges are more likely to disengage from academic activities and may even discontinue their studies altogether [9].

In particular, depressive symptoms have been found to correlate with various maladaptive behaviors among pharmacy students. These include a higher likelihood of engaging in risky behaviors such as illicit substance use and frequent binge drinking [10], as well as reduced participation in physical activity [11]. Moreover, students reporting depressive symptoms have been reported to demonstrate a greater propensity for self-injurious behaviors and suicidal ideation, further underscoring the seriousness of mental health deterioration in this population [5]. In summary, the increasing prevalence of depressive symptoms among pharmacy students has far-reaching implications, not only compromising individual student well-being and academic progression but also adversely affecting institutional outcomes and broader societal health. These findings highlight the urgent need for evidence-based strategies to promote mental health awareness, prevention, and interventions in pharmacy education [12].

A recent systematic review that examined the prevalence and incidence of depressive symptoms among pharmacy students reported considerable variability across studies. The estimated prevalence rates for overall, mild, moderate, and severe depressive symptoms ranged from 4.8% to 78.8%, 9.1% to 42.1%, 5.8% to 30.0%, and 0% to 50.0%, respectively [4]. This 2023 systematic review synthesized 19 studies published between 2009 and January 2022 [4]; however, more than 10 additional studies have been published since 2022. Furthermore, the present meta-analysis incorporates meta-regression to explore associations between prevalence estimates and relevant contextual factors, providing a deeper understanding beyond pooled estimates alone.

This wide range reflects the significant heterogeneity in the study populations, diagnostic criteria, and assessment methodologies. Understanding the pooled prevalence of mental health conditions among pharmacy students, whether overall or stratified by symptom severity, is essential for developing effective prevention strategies, optimizing treatment approaches, and implementing appropriate psychosocial support interventions. Accordingly, the present study aimed to conduct a systematic review and meta-analysis of published literature to determine the pooled prevalence estimates of depression, anxiety, stress, and suicidal ideation among undergraduate pharmacy students.

To this end, a comprehensive systematic review and meta-analysis were undertaken to synthesize the existing evidence from studies reporting the prevalence of depression-related outcomes in this population. The primary focus was to quantify the burden of mental health issues and provide data-driven insights to guide academic institutions and healthcare stakeholders in enhancing mental health initiatives for students.

## 2. Materials and Methods

**Figure 1:**
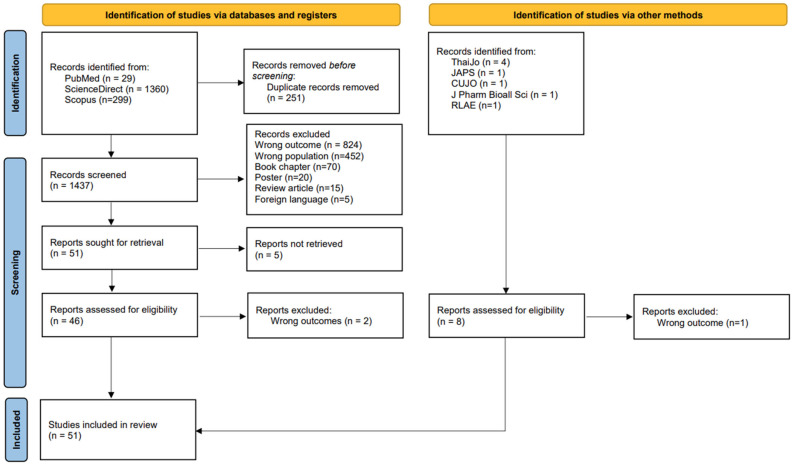
PRISMA flow diagram illustrating the selection process of studies included in the systematic review.

This systematic review and meta-analysis were conducted in accordance with the methodological guidelines outlined in the Cochrane Handbook for Systematic Reviews of Interventions [13]. The review adhered to the Preferred Reporting Items for Systematic Reviews and Meta-Analyses (PRISMA) statement to ensure transparency and methodological rigor, and followed the checklist (Supplementary Material). The review protocol was prospectively registered in the PROSPERO database (Registration No. CRD420251018051), reinforcing the commitment of this study to methodological integrity and reproducibility. A detailed overview of the literature selection process is presented in the PRISMA flow diagram (Figure 1).

### 2.1. Data Sources and Search Strategy

A comprehensive literature search was conducted across the following databases from their inception to 30 April 2025: PubMed, ScienceDirect, Scopus, ThaiJo, and Thai Thesis. Studies published in English or Thai were considered eligible for inclusion. The search strategy employed a combination of controlled vocabulary and free-text terms related to mental health, specifically (Depression OR “Depressive illness” OR “Depressive disorder” OR Depres*) OR (Anxiety) OR (Stress) OR (Suicide OR “Suicidal ideation”) AND (“Pharmacy student” OR “Pharmacy school”). No restrictions were imposed on the publication year to ensure comprehensive coverage of the literature. To promote methodological transparency and reproducibility, the full search strategy applied to each database is provided in Appendix A.1.

### 2.2. Selection Criteria

Studies were included in this review based on the following eligibility criteria: (1) the study investigated the prevalence of mental health problems among pharmacy students; (2) the publication was available in either Thai or English; and (3) the study was presented in the form of an original research article, dissertation, research report, or randomized controlled trial (RCT).

Studies were excluded if they met any of the following criteria: (1) duplicate publication, (2) full-text articles that were inaccessible, or (3) a narrative review or review article.

### 2.3. Data Extraction

All literature retrieved from the electronic databases was imported and screened using the Rayyan platform (https://rayyan.qcri.org/) (accessed on 15 October 2025) for systematic review management. Both electronic and manual search methods were employed to ensure comprehensive identification of eligible studies. Study selection and quality assessment were independently conducted by two reviewers (WP and CT) using reference and citation management programs to facilitate the screening process. Duplicate records were identified and excluded before screening. Titles and abstracts of the remaining studies were reviewed to determine their relevance. In cases where discrepancies arose between the reviewers regarding study inclusion, a consensus was reached through discussion. If necessary, the full text was retrieved to resolve disagreements. When a consensus could not be reached, a third reviewer (KS) was consulted to provide a final decision.

Studies that met the inclusion criteria based on title and abstract screening were further assessed through full-text review. Eligibility was confirmed according to predefined inclusion and exclusion criteria, and detailed reasons for exclusion were documented. For studies with inaccessible full texts, the corresponding authors were contacted to obtain the necessary data. Studies were excluded if the full text could not be obtained or if no response was received.

The study selection process, including the number of records identified, screened, included, and excluded along with the corresponding reasons, was documented using the PRISMA flow diagram framework. Key data from each included study were systematically extracted using a standardized data extraction form adapted from the Cochrane Data Collection Form and recorded in a Microsoft Excel spreadsheet for analysis. In instances where the data formats were unclear or interpretation was uncertain, a third reviewer (KS) was consulted. In addition, where necessary, the authors of the original studies were contacted for clarification or to obtain missing information.

### 2.4. Quality Assessment

The quality of non-randomized studies was assessed using the Newcastle–Ottawa Scale (NOS), which assigns scores ranging from 0 to 10. This tool evaluates studies across three key domains: (1) selection of study groups, (2) comparability of groups, and (3) outcome ascertainment. Based on the NOS scoring, studies were classified as follows: scores <3 were considered low quality, those between 4 and 6 as moderate quality, and those between 7 and 10 as high quality [14].

For RCTs, the study quality was assessed using both the Jadad Scale and the Cochrane Risk of Bias tools. According to the Jadad Scale, studies scoring between 3 and 5 points were deemed to be of high quality, while those with scores ≤2 were classified as low quality [15]. The Risk of Bias tool evaluated the potential for bias within each domain, with ratings as follows: Low Risk of Bias indicates minimal concerns regarding bias in that domain; Some concerns suggest possible Risk of Bias; and High Risk of Bias indicates clear evidence of bias [16].

The Grading of Recommendations Assessment, Development, and Evaluation (GRADE) approach was applied as the final step in the quality assessment process to strengthen the credibility and interpretability of the study findings. The GRADE framework evaluates the certainty of evidence across five critical domains: (1) Risk of bias, which assesses the methodological rigor and potential for systematic errors within individual studies; (2) Inconsistency, which examines the degree of heterogeneity in outcomes across multiple studies; (3) Indirectness, which evaluates the extent to which the evidence is directly applicable to the population, intervention, comparator, and outcomes of interest in real-world clinical settings; (4) Imprecision, which considers the level of uncertainty around the effect estimates, often influenced by sample size and confidence interval width; and (5) Other considerations, including publication bias, residual confounding, and the magnitude or dose–response gradient of the observed effect. Based on an evaluation of these domains, the GRADE system categorizes the certainty of evidence into four levels: (1) High certainty, indicating strong confidence that the true effect is close to the estimated effect; (2) Moderate certainty, suggesting that the true effect is likely close to the estimate, although there is a possibility that it may differ substantially; (3) Low certainty, reflecting limited confidence in the estimate, with a substantial likelihood that the true effect may differ; and (4) Very low certainty, implying that the estimate is highly uncertain and that the true effect is likely to differ significantly from the reported value [17].

### 2.5. Statistical Analysis

A meta-analysis was conducted for each mental health outcome using STATA statistical software version 15 (StataCorp, College Station, TX, USA). Pooled prevalence estimates were calculated using a random-effects model to account for inter-study variability, and the results are reported as effect sizes with corresponding 95% confidence intervals (CIs). Moreover, 95% prediction intervals (PIs) were calculated for all pooled estimates, which accounted for an estimated range for the true treatment effect in an individual study and the expected uncertainty of the estimate in a new study [18]. Forest plots were generated to visually present the combined results. Heterogeneity across studies was assessed using the *I*^2^ statistic, with values exceeding 50% and a *p*-value < 0.05 indicating substantial heterogeneity. Inter-rater agreement between the independent reviewers involved in study selection and the risk of bias assessment was evaluated using Cohen’s kappa (κ) coefficient to quantify the level of concordance [19].

Sensitivity analyses were conducted to assess the robustness of the primary findings by reestimating the pooled prevalence using a fixed-effects model. Subgroup analyses were stratified by the WHO regional classification (Africa, Americas, South-East Asia, Europe, Eastern Mediterranean, and Western Pacific), World Bank income group (low-, lower-middle-, upper-middle-, and high-income), and average gross domestic product (GDP). To further explore the sources of heterogeneity, meta-regression analyses were performed to evaluate the impact of geographical region, income classification, and average GDP on the pooled prevalence estimates of mental health symptoms.

Assessment of potential publication bias was undertaken through visual inspection of funnel plots when a sufficient number of studies (typically ≥10) were available. Statistical tests for funnel plot asymmetry, including Begg’s and Egger’s tests, were applied, with a *p*-value < 0.10 considered indicative of publication bias [20,21]. In cases where publication bias was detected, the trim-and-fill method was employed to adjust for potential bias and provide corrected estimates [22].

## 3. Results

A total of 1688 records were initially identified through database searches, comprising 29 studies from PubMed, 1360 from ScienceDirect, and 299 from Scopus. After removing 251 duplicate records, 1437 unique studies were screened. Screening of titles and abstracts led to the exclusion of 824 studies that were deemed irrelevant to the research objectives.

Further exclusions were made based on the following criteria: 452 studies involved non-target populations, 70 were book chapters, 20 were poster presentations, 15 were peripheral articles, and five were published in non-English or non-Thai languages. An additional five studies were excluded after full-text screening owing to outcome irrelevance, and two were excluded owing to insufficient alignment with the study scope. Supplementary searches yielded eight additional studies from other sources, including ThaiJo (*n* = 4), Journal of Advanced Pharmaceutical Sciences (JAPS; *n* = 1), Chulalongkorn University Journal of Opinion (CUJO; *n* = 1), Journal of Pharmacy and Bioallied Sciences (*n* = 1), and Revista Latino-Americana de Enfermagem (RLAE; *n* = 1). One of these studies was excluded because of a lack of relevance. Finally, 51 studies met the inclusion criteria and were included in the final analysis. The study selection process is illustrated in Figure 1.

### 3.1. Characteristics of Included Studies

A total of 51 studies published between 2005 and 2024, which encompassed data from 17,717 participants across 16 countries, were included in this review. These countries included the United States, Thailand, Brazil, Malaysia, Syria, Pakistan, Poland, France, Portugal, Nigeria, Saudi Arabia, Sudan, Lebanon, Egypt, the United Arab Emirates, and Vietnam. The distribution of studies by country was as follows: the United States (*n* = 11), Thailand (*n* = 8), Brazil (*n* = 3), Malaysia (*n* = 8), Syria (*n* = 1), Pakistan (*n* = 3), Poland (*n* = 1), France (*n* = 2), Portugal (*n* = 2), Nigeria (*n* = 1), Saudi Arabia (*n* = 2), Sudan (*n* = 4), Lebanon (*n* = 1), Egypt (*n* = 2), the United Arab Emirates (*n* = 2), and Vietnam (*n* = 1).

The participants were pharmacy students aged 18–34 years. Various validated screening tools were used to assess mental-health outcomes in the reviewed studies. These included the following: the Diagnostic and Statistical Manual of Mental Disorders (DSM), Beck Depression Inventory (BDI), Patient Health Questionnaire (PHQ), Center for Epidemiological Studies–Depression Scale (CES-D), Kutcher Adolescent Depression Scale (KADS), Hospital Anxiety and Depression Scale (HADS), Depression Anxiety Stress Scale (DASS), Suanprung Stress Test-20 (SPST-20), State-Trait Anxiety Inventory (STAI), Westside Test Anxiety Scale (WTAS), Generalized Anxiety Disorder-7 (GAD-7), Zung Self-Rating Anxiety Scale (SAS), Cognitive Test Anxiety Scale–2nd Edition (CTAS-2), Counseling Center Assessment of Psychological Symptoms-62 (CCAPS-62), Test Anxiety Inventory (TAI), Beck Scale for Suicidal Ideation (BSI), Stress Test Questionnaire (ST-5), and the Perceived Stress Scale (PSS). In some studies, the specific tools used for screening were not reported.

The majority of included studies employed a cross-sectional design (*n* = 50), while one study was a randomized controlled trial. The primary mental health outcomes assessed were depression, anxiety, suicidal ideation, and stress. A summary of the study characteristics and outcome measures is presented in Table 1.

### 3.2. Quality Assessment of Included Studies

The quality assessment of cross-sectional, longitudinal, and repeated-measures studies conducted using the NOS revealed that all 48 studies achieved scores ranging from 7 to 9, indicating a high level of methodological quality. Two longitudinal and repeated-measures studies were evaluated using the same NOS criteria. These studies received scores ranging from 6 to 7, which correspond to a moderate to high level of quality. The detailed results are presented in Table 2. The single RCT included in this review was assessed using the Risk of Bias tool. The results indicate a high risk of bias, demonstrating clear methodological concerns (Table 3).

### 3.3. Prevalence of Depression Among Pharmacy Students

**Figure 2:**
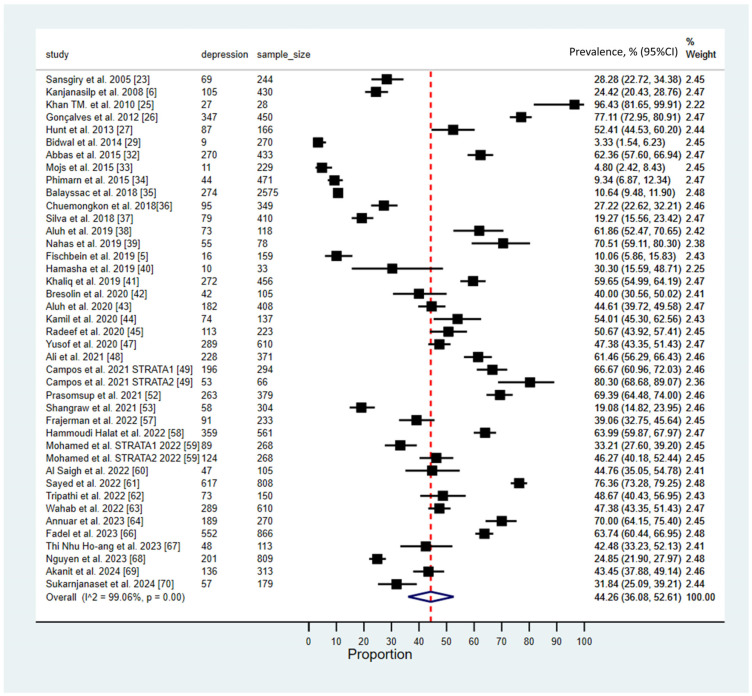
Pooled prevalence of depression among pharmacy [5,6,23,25,26,27,29,32,33,34,35,36,37,38,39,40,41,42,43,44,45,47,48,49,52,53,57,58,59,60,61,62,63,64,66,67,68,69,70]. (The solid black square (■) represents the point estimate of the observed outcome. The diamond square (◆) denotes the pooled or overall estimate derived from the aggregated data. The dotted line (---) indicates the final pooled estimate).

The meta-analysis of depression prevalence among pharmacy students revealed a pooled prevalence estimate of 44.26% (95% CI: 36.08–52.61 and 95% PI: 8.6% to 85.3%), indicating a substantial burden within this population. The analysis demonstrated considerable heterogeneity across studies (*I*^2^ = 99.06, *p* < 0.001). A forest plot summarizing these findings is shown in Figure 2.

### 3.4. Prevalence of Anxiety Among Pharmacy Students

**Figure 3:**
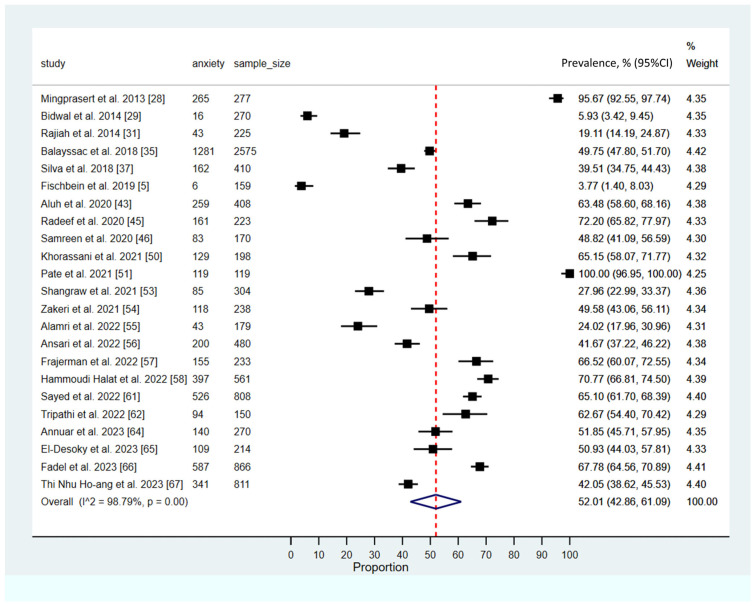
Pooled prevalence of anxiety among pharmacy students [5,28,29,31,35,37,43,45,46,50,51,53,54,55,56,57,58,61,62,64,65,66,67]. (The solid black square (■) represents the point estimate of the observed outcome. The diamond square (◆) denotes the pooled or overall estimate derived from the aggregated data. The dotted line (---) indicates the final pooled estimate).

The meta-analysis assessing the prevalence of anxiety among pharmacy students revealed a pooled prevalence estimate of 52.01% (95% CI: 42.86–61.09; 95% PI: 19.3% to 80.5%), indicating a high burden of anxiety within this population. The analysis revealed substantial heterogeneity across the included studies (*I*^2^ = 98.79, *p* < 0.001). These findings are depicted in Figure 3.

### 3.5. Prevalence of Stress Among Pharmacy Students

**Figure 4:**
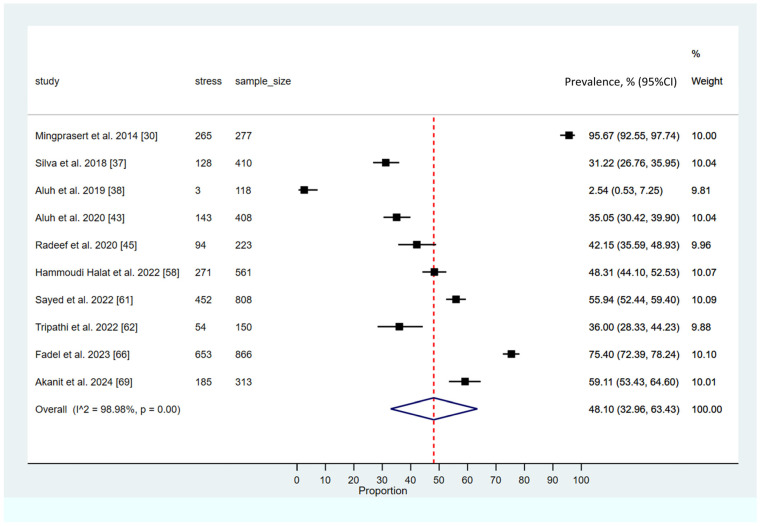
Pooled prevalence of stress among pharmacy students [30,37,38,43,45,58,61,62,66,69]. (The solid black square (■) represents the point estimate of the observed outcome. The diamond square (◆) denotes the pooled or overall estimate derived from the aggregated data. The dotted line (---) indicates the final pooled estimate).

The meta-analysis examining the prevalence of stress among pharmacy students yielded a pooled prevalence estimate of 48.10% (95% CI: 32.96–63.43; 95% PI: 12.9% to 86.5%), highlighting a considerable psychological burden within this population. The analysis demonstrated a high degree of heterogeneity across the included studies (*I*^2^ = 98.98, *p* < 0.001). A visual summary of these findings is presented in Figure 4.

### 3.6. Prevalence of Suicidal Ideation Among Pharmacy Students

**Figure 5:**
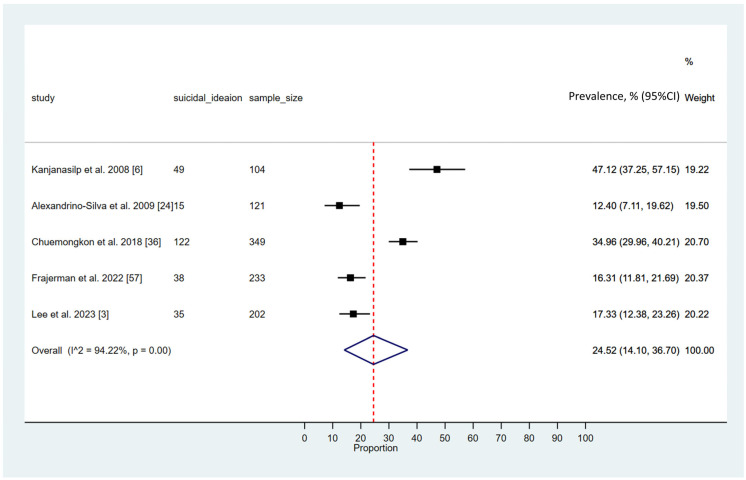
Pooled prevalence of suicidal ideation among pharmacy students [3,6,24,36,57]. (The solid black square (■) represents the point estimate of the observed outcome. The diamond square (◆) denotes the pooled or overall estimate derived from the aggregated data. The dotted line (---) indicates the final pooled estimate).

The meta-analysis assessing the prevalence of suicidal ideation among pharmacy students revealed a pooled prevalence of 24.52% (95% CI: 14.10–36.70; 95% PI: 3.6% to 72.2%), indicating a concerning level of psychological distress in this population. The analysis demonstrated substantial heterogeneity among the included studies (*I*^2^ = 94.22, *p* < 0.001). These findings are depicted in Figure 5.

### 3.7. Subgroup Analysis

Subgroup analysis revealed significant regional variations in the pooled prevalence of depression among pharmacy students across WHO regions. The highest prevalence was observed in the Western Pacific Region at 62.60% (95% CI: 52.91–71.81, *I*^2^ = 95.94%, *p* < 0.001), followed by the Eastern Mediterranean Region at 54.05% (95% CI: 46.15–61.85, *I*^2^ = 95.83%, *p* < 0.001), and the African Region at 48.48% (95% CI: 44.21–52.78). The South-East Asia Region and the Region of the Americas reported comparable prevalence estimates of 33.13% (95% CI: 16.45–52.33, *I*^2^ = 98.79%, *p* < 0.001) and 33.73% (95% CI: 19.08–50.16, *I*^2^ = 98.44%, *p* < 0.001), respectively. The lowest prevalence was reported in the European Region at 16.64% (95% CI: 7.13–29.08, *I*^2^ = 97.73%, *p* < 0.001) (Appendix A.2), underscoring substantial heterogeneity in depression prevalence across global regions.

Similarly, considerable regional variation was observed in the prevalence of anxiety. The South-East Asia Region reported the highest rate at 95.67% (95% CI: 92.55–97.74), followed by the African Region at 63.48% (95% CI: 58.60–68.16) and the Eastern Mediterranean Region at 54.61% (95% CI: 43.39–65.61, *I*^2^ = 97.44%, *p* < 0.001). The European Region reported an anxiety prevalence of 51.75% (95% CI: 40.29–63.11), while lower estimates were found in the Western Pacific Region (47.20%; 95% CI: 18.52–76.93) and the Region of the Americas (43.30%; 95% CI: 22.44–65.48, *I*^2^ = 99.10%, *p* < 0.001) (Appendix A.3). These results reflect the marked differences in the prevalence of anxiety among pharmacy students across geographical contexts. In terms of suicidal ideation, the highest pooled prevalence was found in the South-East Asia Region at 37.66% (95% CI: 33.24–42.20), indicating a notable mental health burden among students in this area. In contrast, substantially lower rates were reported in the Region of the Americas at 15.39% (95% CI: 11.63–19.57) and the European Region at 16.31% (95% CI: 11.81–21.69) (Appendix A.4), further highlighting regional disparities in mental health outcomes among pharmacy students.

### 3.8. Meta-Regression

**Figure 6:**
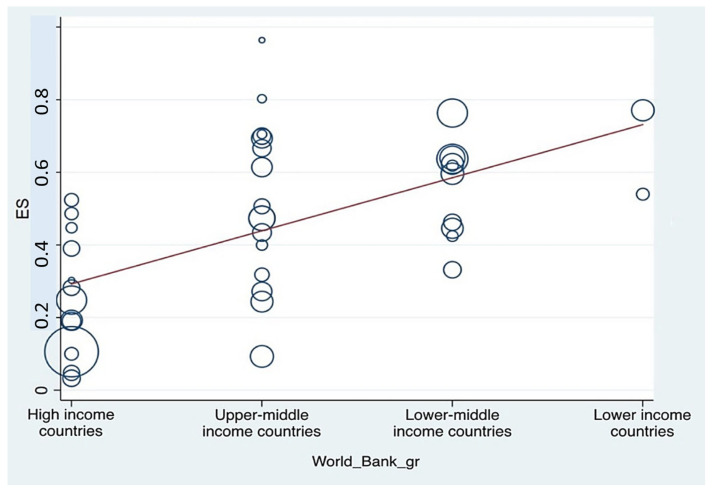
Meta-regression analysis assessing the association between the prevalence of depression among pharmacy students and national income level as classified by the World Bank. (The circle represents an individual study, with its size proportional to its statistical weight in the meta-regression model. The solid red line shows the fitted meta-regression line, indicating the estimated linear association between the covariate and the outcome).

**Figure 7:**
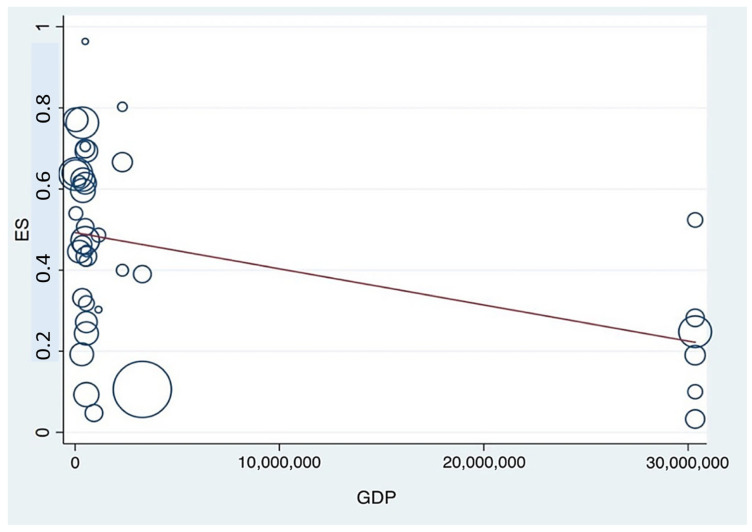
Meta-regression analysis assessing the association between depression prevalence among pharmacy students and national GDP. (The circle represents an individual study, with its size proportional to its statistical weight in the meta-regression model. The solid red line shows the fitted meta-regression line, indicating the estimated linear association between the covariate and the outcome).

This study employed meta-regression analysis to investigate the association between depression prevalence among pharmacy students and three key country-level variables: WHO regional classification, World Bank income level, and GDP. The analysis of the relationship between national income level and depression prevalence revealed a statistically significant positive association, with lower-income countries reporting higher prevalence rates. The meta-regression yielded τ^2^ = 0.027, 95% CI: 0.0800–0.2123, *I*^2^ = 88.84%, and *p* < 0.001. These findings underscore the influence of socioeconomic disparities on mental health outcomes among student populations. A graphical representation of this association is presented in Figure 6. Similarly, a statistically significant negative association was observed between national GDP and the prevalence of depression. The analysis produced τ^2^ = 0.0362, 95% CI: –1.5 × 10^−8^ to –2.83 × 10^−9^, *I*^2^ = 94.75%, and *p* = 0.005, indicating that countries with lower GDP tend to have a higher prevalence of depression among pharmacy students. This relationship is illustrated in Figure 7.

**Figure 8:**
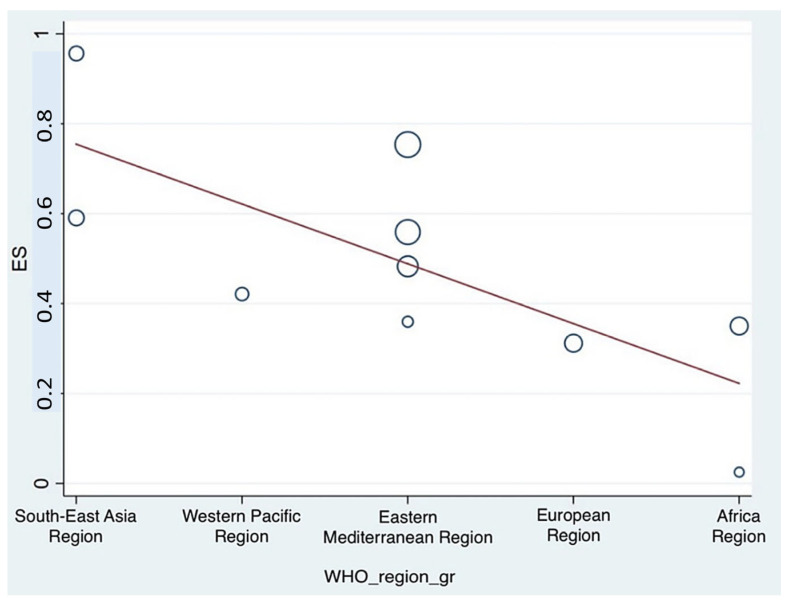
Meta-regression analysis results following examination of the association between the prevalence of stress among pharmacy students and the WHO regional classification. (The circle represents an individual study, with its size proportional to its statistical weight in the meta-regression model. The solid red line shows the fitted meta-regression line, indicating the estimated linear association between the covariate and the outcome).

In addition, a meta-regression analysis assessing the association between WHO regional classification and the prevalence of stress among pharmacy students revealed a statistically significant negative relationship. The results showed τ^2^ = 0.0283, 95% CI: –0.2311 to –0.0348, *I*^2^ = 91.48%, and *p* = 0.014, suggesting that regional characteristics as classified by the WHO may significantly influence stress prevalence in this population. A visual summary of these findings is depicted in Figure 8.

### 3.9. Publication Bias

**Figure 9:**
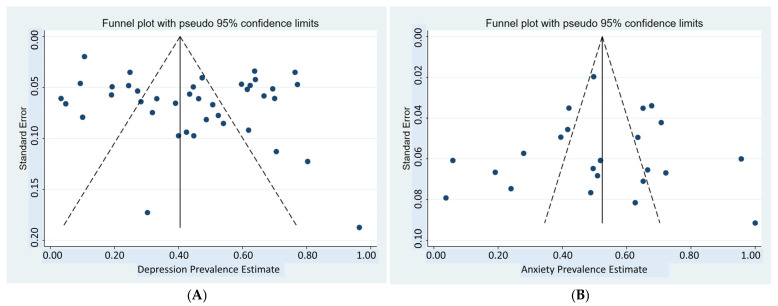

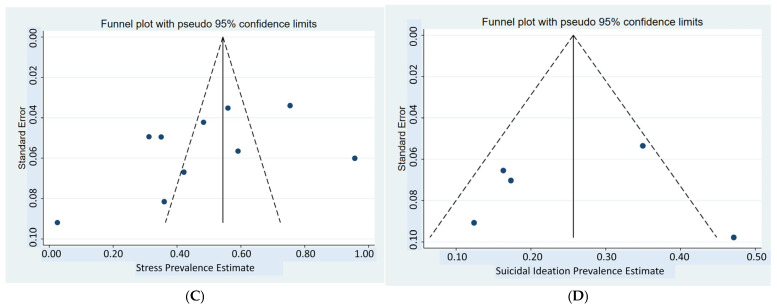
(**A**) Forest plot for the subgroup analysis of depression prevalence. (**B**) Forest plot for the subgroup analysis of anxiety prevalence. (**C**) Forest plot for the subgroup analysis of stress prevalence. (**D**) Forest plot for the subgroup analysis of suicidal ideation prevalence. (Each point represents an individual study in the meta-analysis. The vertical line indicates the pooled overall effect, and the diagonal lines form a symmetrical funnel corresponding to the expected 95% confidence limits in the absence of publication bias).

To examine the potential for publication bias, which is defined as the systematic tendency to publish studies reporting favorable or hypothesis-confirming results more frequently, this study utilized multiple diagnostic techniques, including visual inspection of funnel plots, Egger’s test, and Egger’s regression plots. For depression prevalence analysis, the funnel plot (Figure 9A) appears symmetrical around the vertical axis, representing the pooled effect size, and indicating no substantial evidence of publication bias. This was corroborated by Egger’s test, which yielded an intercept of 0.39 (SE = 0.24, 95% CI: −0.09 to 0.88, *t* = 1.63, *p* = 0.11), suggesting the absence of statistically significant bias. The Egger’s regression plot further supported this finding, with the regression line intersecting the Y-axis near zero and the 95% CI spanning across zero.

A similar pattern was observed in anxiety prevalence. The funnel plot (Figure 9B) shows visual symmetry around the central vertical axis, suggesting no evidence of publication bias. Egger’s test confirmed this interpretation, with an intercept of –0.35 (SE = 0.58, 95% CI: –1.56 to 0.85, *t* = –0.62, *p* = 0.54). Additionally, the Egger’s regression plot demonstrated a Y-axis intersection near zero and a 95% CI encompassing zero, indicating no statistical evidence of publication bias in the included studies. In contrast, the analysis of stress prevalence revealed potential publication bias. Although the funnel plot (Figure 9C) appears visually symmetrical, Egger’s test indicated statistically significant asymmetry, with an intercept of –2.21 (SE = 0.93, 95% CI: –4.37 to –0.06, *t* = –2.37, *p* = 0.045). This finding was further supported by Egger’s regression plot, which showed the regression line intersecting the Y-axis near zero, but with a 95% CI that did not span zero, providing additional evidence of publication bias in the included studies. No evidence of publication bias was observed in the analysis of suicidal ideation prevalence. The funnel plot (Figure 9D) is symmetrical, and Egger’s test yielded non-significant results (intercept = 0.21 SE = 1.39, 95% CI: –4.23 to 4.65, *t* = 0.15, *p* = 0.889). Similarly, Egger’s regression plot showed a regression line intersecting the Y-axis near zero with a 95% CI crossing zero, supporting the absence of publication bias.

## 4. Discussion

A review of the literature revealed that the present study is an updated systematic review and meta-analysis that specifically examined the prevalence of mental health problems, namely depression, anxiety, suicidal ideation, and stress, among pharmacy students globally. The findings of this study indicated that the pooled prevalence of depression was 44.26%, anxiety 52.01%, stress 48.10%, and suicidal ideation 24.52%. These results provide compelling evidence supporting the urgent need to develop mental health support strategies tailored for pharmacy students. Such strategies are essential for formulating policies and practices aimed at promoting student well-being and mitigating the impact of mental health problems within this population.

The quality of the included studies was assessed using the NOS and the Risk of Bias Tool to ensure data reliability and methodological rigor. Among the 48 included cross-sectional studies, most received NOS scores ranging from 7 to 9, indicating high methodological quality. This reflects a robust study design and reliable data collection procedures. Two longitudinal and repeated-measures studies scored between 6 and 7, indicating moderate-to-high quality, although some methodological limitations were noted. The single RCT included in this review was evaluated using the Risk of Bias Tool. Overall, the included studies were of high quality and provided reliable evidence. Nevertheless, it is important to interpret findings from studies with a higher risk of bias cautiously to avoid overestimation or misinterpretation of the results. Therefore, the present study provides valuable high-quality data that can inform the development of targeted and effective mental health interventions for pharmacy students.

When compared with previous research, the prevalence of depression (44.26%) and suicidal ideation (24.52%) among pharmacy students observed in this study appears substantially higher than those reported among medical students. For instance, a meta-analysis by Rotenstein et al. (2016) [71] found that the prevalence of depression and suicidal ideation among medical students was 27.2% and 11.1%, respectively. Furthermore, the highest prevalence of depression among pharmacy students in this study was found in the Western Pacific Region, which contrasts with the findings of Shorey et al. (2022) [72]. Their global meta-analysis of depression in adolescents reported the highest prevalence in Asia, the Middle East, and Africa.

The subgroup analysis revealed a substantial disparity in depression prevalence between the Western Pacific Region (62.60%) and the European Region (16.64%). This difference may reflect the tendency for pharmacy and other health-science students in Asian contexts to report higher levels of psychological distress, a phenomenon often linked to more intensive academic workloads, competitive learning environments, and cultural stigma associated with seeking mental-health support, which may delay early intervention and exacerbate symptoms [69,73,74]. Conversely, European higher-education settings generally offer more established student mental-health support systems, greater openness toward mental-health disclosure, and wider availability of institutional counseling services, potentially contributing to lower observed prevalence [75,76]. Furthermore, variation in screening instruments, diagnostic thresholds, and symptom reporting norms across regions may also influence prevalence estimates and contribute to observed discrepancies between studies conducted in Europe and Asia.

The included studies utilized a range of screening instruments to assess depression, anxiety, and stress (e.g., BDI, PHQ-9, DASS-21), each applying different scoring thresholds and severity classifications. In the present meta-analysis, prevalence was extracted based on the proportion of participants meeting the minimum cut-off for clinical symptoms, irrespective of severity level, as defined in the original studies. Consequently, the pooling of outcomes derived from heterogeneous measurement tools and varying severity thresholds may have contributed to the high heterogeneity observed, potentially resulting in an overestimation of prevalence in the aggregated meta-analytic estimates.

This study also identified significant associations between national economic indicators and depression prevalence. Specifically, countries with lower income levels had a significantly higher prevalence of depression among pharmacy students. A similar inverse association was observed between GDP and the prevalence of depression, suicidal ideation, and stress, indicating that students in countries with lower GDPs are more likely to experience these mental health challenges. These findings are inconsistent with a systematic review and meta-analysis by Akhtar et al. (2020) [77], which assessed the prevalence of depression among university students in low- and middle-income countries (LMICs). Their review of 37 studies involving 76,608 students across 20 countries concluded that there were no significant differences in depression prevalence based on study design, sampling method, sample size, field of study, academic level, economic region, or screening tool.

Our presented association, therefore, warrants further examination to understand the potential causal pathways linking macroeconomic conditions to the psychological well-being of student populations. There are several potential mechanisms through which national economic indicators may influence mental health outcomes among students. First, economic hardship at the national level often translates into constrained public investment in education and mental health care infrastructure. In LMICs, limited access to psychological services, financial aid, and academic support programs can exacerbate students’ vulnerability to stress and depression [78]. Second, macroeconomic instability may lead to household-level financial insecurity, increasing the pressure on students who must simultaneously cope with academic demands and socioeconomic stressors [79]. This aligns with social causation theory, which posits that poverty and low socioeconomic status contribute causally to the onset of mental disorders, particularly depression [80].

In the educational context, pharmacy students in LMICs may face heightened academic workloads, poor educational infrastructure, and limited employment prospects after graduation, all of which may synergistically compound psychological distress. Recent data from LMICs have shown that pharmacy curricula are perceived as academically intense and often lack adequate support systems and career counseling services [5,81]. These factors contribute to increased rates of anxiety and depression compared to students in higher-income settings, where institutional support and resilience-building programs are more accessible [82].

Interestingly, the current findings diverge from those reported by Akhtar et al. (2020) [77], whose systematic review and meta-analysis of 37 studies involving over 76,000 university students across 20 LMICs found no significant differences in depression prevalence based on economic region or other study characteristics. This discrepancy may have arisen from several methodological factors. For example, Akhtar et al. aggregated data across diverse academic disciplines and educational contexts, potentially diluting discipline-specific stressors that are particularly pronounced in fields such as pharmacy. Moreover, variations in the operationalization of depression, cultural norms regarding emotional disclosure, and differences in measurement tools may further obscure the relationship between economic indicators and the prevalence of depression [83].

The high heterogeneity observed in our meta-analysis may influence the reliability and stability of the association between GDP classification and depression prevalence. Previous meta-analyses demonstrated that heterogeneity is often substantial in mental-health prevalence research, and high *I*^2^ can affect the precision of meta-regression coefficients, potentially leading to over- or under-estimation of socioeconomic effects [84,85]. Moreover, socioeconomic gradients in mental health are multifactorial, influenced not only by GDP but also by health system capacity, cultural perceptions, and stress-coping environments [78,86,87].

Another possible explanation is the heterogeneity of LMICs. When grouped collectively, countries classified as LMICs exhibit wide variations in healthcare access, cultural attitudes toward mental health, and economic resilience, which could differentially affect student well-being. For instance, studies in sub-Saharan Africa have reported a higher prevalence of depression than those in Southeast Asia, even among countries with comparable GDP per capita, indicating the influence of additional sociopolitical and cultural factors [88,89].

The observed association between low national income and a high prevalence of depression underscores the need for targeted mental health interventions in economically disadvantaged regions. Strategies may include the integration of mental health services within academic institutions, expansion of financial support for students, and structural investments in the health and education sectors. Future research should employ multilevel modeling approaches to disentangle individual-level stressors from country-level economic variables and identify the protective factors that buffer mental distress in low-resource settings.

Although the present study did not specifically analyze the causal factors underlying depression, anxiety, suicidal ideation, or stress, the existing literature provides important insights into the potential mechanisms that contribute to these mental health issues among pharmacy students. Previous empirical studies identified psychosocial and academic stressors as key contributors, suggesting a complex interplay between personal, interpersonal, and institutional factors. A descriptive cross-sectional study by Sukarnjanaset et al. (2024) [70] revealed that interpersonal relationships with peers, romantic partners, and/or family members were significantly associated with depressive symptoms in pharmacy students. These difficulties can foster feelings of loneliness, rejection, and low self-worth, which can precipitate or exacerbate depressive episodes [90]. Moreover, sleep disturbances, which were also highlighted in the study by Sukarnjanaset [70], are both a symptom of and a potential causal factor for depression. Insufficient or poor sleep quality has been shown to impair emotional regulation and cognitive performance, thereby increasing susceptibility to mood disorders [91,92]. Another study also noted that students in their first year of pharmacy school experienced higher levels of depression than their senior peers [93], possibly because of academic transition stress, lack of coping mechanisms [94], and diminished social support during the adjustment phase [95,96].

Complementing the above findings, Akanit et al. (2024) [69] utilized a mixed-methods approach combining quantitative surveys and qualitative interviews to explore academic-related stressors. They identified five primary academic challenges that are strongly associated with depression: (1) unmet academic expectations, (2) difficulty keeping pace with classmates, (3) overly complex course content, (4) insufficient time for exam preparation, and (5) presentation-related anxiety. These findings align with the academic stress model, which posits that a mismatch between academic demands [97] and students’ perceived capabilities trigger psychological strain [98,99]. When students are unable to meet their academic goals, either self-imposed or externally driven, they may experience feelings of inadequacy, fear of failure, and learned helplessness, all of which are key precursors of depression [100,101].

Moreover, the pharmacy curriculum is widely acknowledged to be academically rigorous and often involves heavy workloads, high-stakes assessments, and competitive environments [102,103]. These factors may disproportionately affect students who lack effective time management or study skills, or those with high levels of perfectionism, a trait associated with increased vulnerability to depressive symptoms [104]. Anxiety around public presentations, a stressor identified by Akanit et al. [69], is particularly relevant for students with social anxiety or low self-esteem and may exacerbate avoidance behaviors and isolation, compounding depressive tendencies [105,106].

Identifying these causative relationships has practical implications. Interventions targeted at improving interpersonal skills, stress management, and sleep hygiene should be integrated into pharmacy education programs. Additionally, academic support services such as tutoring, peer mentoring, and flexible examination scheduling may mitigate the negative mental health impacts of educational stressors. From a research perspective, longitudinal studies are needed to establish the temporal relationship between these stressors and mental health outcomes. Moreover, mediation analyses could elucidate how variables such as resilience, coping style, and social support modulate the effects of these stressors on depressive symptoms.

Based on subgroup analyses, our findings highlight the need for tiered, context-specific mental health interventions for pharmacy students. In low- and middle-income settings with higher prevalence, priorities include improving access to basic mental health services, early screening, and referral pathways. In high-income settings, interventions should focus on academic stress management, workload optimization, and resilience building. Stage-specific support, including transition programs for junior students and stress-coping interventions for senior students, is also warranted.

High heterogeneity is common in meta-proportion analyses due to methodological and contextual diversity across studies, particularly in mental-health research [107]. Potential contributors include variation in assessment instruments and diagnostic cut-offs such as the use of DASS-21, PHQ-9, GAD-7, HADS, and Beck inventories each demonstrating different sensitivity and scoring thresholds that influence prevalence estimates [108,109]. Considerable variation in population characteristics, academic stage, and learning environment (e.g., pre-clinical vs. clinical years, examination periods, internship demands) further contributes to psychological burden disparities among pharmacy students [110]. Additionally, geographical and sociocultural differences, including mental-health stigma, access to psychological support, and variability in educational systems, have been shown to influence prevalence outcomes across countries [86,111]. Temporal factors also play an important role, as studies conducted during the COVID-19 pandemic reported significantly higher levels of anxiety and depression than pre-pandemic periods [112,113]. Heterogeneity is further amplified by differences in study design, sampling approaches (online vs. campus-based surveys), voluntary participation, and sample size variation, with smaller studies tending to overestimate prevalence in meta-analyses. Moreover, the statistical behavior of proportional data, particularly when estimates approach 0% or 100%, naturally increases variance across studies [71,114].

This study has several limitations that warrant consideration. First, only one RCT was included in the analysis, and it was assessed as having a high risk of bias using the Risk of Bias Tool, which may undermine the overall credibility of the pooled results. Second, the included studies were conducted across diverse countries with differing cultural norms, educational structures, and social contexts, which likely contributed to substantial variability in reported outcomes. The extremely high heterogeneity observed across all pooled prevalence estimates (*I*^2^ > 98%) and the very wide 95% PIs of all outcomes indicates substantial between-study heterogeneity and considerable uncertainty regarding the expected prevalence in a future study. Therefore, these findings have important implications for their interpretation. In meta-analyses of proportions, such heterogeneity reflects genuine between-study differences arising from variation in population characteristics, geographic settings, assessment instruments, cut-off thresholds, and study designs. Although random-effects models were appropriately applied, the resulting pooled prevalence estimates should be interpreted primarily as descriptive summaries of overall burden rather than precise epidemiological estimates applicable to specific populations. Consequently, these pooled estimates may have limited statistical and clinical precision, and their generalizability is inherently constrained. The prevalence figures reported in this study are thus intended to illustrate broad global trends in mental health burden among pharmacy students, while subgroup and meta-regression analyses should be viewed as exploratory and hypothesis-generating. Furthermore, the absence of data from some countries with distinct mental health profiles may have further limited the consistency and generalizability of the findings. Third, the use of a wide range of mental health assessment tools, such as the PHQ-9, BDI, HADS, DASS-21, and ST-5, may have introduced variability in measurement sensitivity and specificity, potentially affecting the comparability of results across studies and contributing to measurement bias. Fourth, this review may be subject to geographic and language bias, as gray literature searches were limited to ThaiJo and Thai Thesis databases and only English- and Thai-language studies were included. Moreover, this study did not include searches in specialized psychiatric and medical databases such as PsycINFO and Embase, which may have resulted in the omission of relevant mental health–related literature. Future research should incorporate PsycINFO and Embase to achieve more comprehensive coverage and reduce the potential for publication bias. Finally, subgroup analyses by WHO region and World Bank income level were performed to explore global geographic and economic patterns in mental health prevalence among pharmacy students. However, some subgroups, particularly the European region, included few studies, which may limit estimate stability and precision. Therefore, these findings should be interpreted cautiously and considered exploratory. The classifications were retained to maintain global relevance and facilitate cross-regional comparison, with results intended to illustrate broad trends rather than definitive regional prevalence.

Future research should investigate the environmental factors that may influence the mental health of pharmacy students, including the structure of the educational system, academic pressure, work–life balance, and social environment, all of which may play critical roles in psychological well-being. Insights from such studies could contribute to a more comprehensive understanding of the determinants of mental health issues in this population. Furthermore, the findings should be applied to inform the development of preventive measures aimed at supporting the mental health of students. This may include the implementation of psychological counseling services, stress management training programs, and the creation of supportive networks within universities to foster resilience and promote overall mental wellness among pharmacy students.

## 5. Conclusions

In conclusion, this systematic review and meta-analysis provide a comprehensive global overview and evidence of the prevalence of mental health problems among pharmacy students. The findings revealed alarmingly high prevalence rates of mental health issues among pharmacy students, including depression, anxiety, stress, and suicidal ideation. This highlights the critical and pervasive burden of psychological distress in this population. Given the demanding nature of pharmacy education and its associated stressors, the mental well-being of pharmacy students warrants immediate and sustained attention from academic institutions, healthcare policymakers, and mental health professionals.

## Figures and Tables

**Table 1:** Summary of general data extracted from all 51 included studies, detailing the study design, geographic location, sample size, participant characteristics, screening tools used, and primary mental health outcomes assessed.

No	Author	Year	Country	Population	Age (Year)	Number of Participants	Tools	Study Design	Outcome
1	Sansgiry et al. [23]	2005	USA	Pharmacy students	27.78 ± 4.06	244	N/A	Cross-sectional study	Depression
2	Kanjanasilp et al. [6]	2008	Thailand	Pharmacy students	20.1 ± 1.7	430	Stress Screening Form of the Department of Mental Health, Ministry of Public Health Suicide Risk Screening Form	Cross-sectional study	Depression, Suicidal ideation
3	Alexandrino-Silva et al. [24]	2009	Brazil	Pharmacy students	20.92 ± 2.207	122	Beck Scale for Suicidal Ideation (BSI)	Cross-sectional study	Suicidal ideation, Depressive
4	Khan TM. et al. [25]	2010	Malaysia	Pharmacy students	19–22	28	DSM IV-TR	Cross-sectional study	Depression
5	Gonçalves et al. [26]	2012	Syria	Pharmacy students	20.03 ± 1.92	262	The Beck Depression Inventory (BDI)	Prospective longitudinal study	Depression
6	Hunt et al. [27]	2013	USA	Pharmacy students	18–23	166	Patient Health Questionnaire-9 (PHQ-9)	A cross-sectional study	Depression
7	Mingprasert et al. [28]	2013	Thailand	Pharmacy students	N/A	277	State Trait Anxiety Inventory (STAI)	Cross-sectional study	Anxiety
8	Bidwal et al. [29]	2014	USA	Pharmacy students	26.4 ± 3.6	309	Fourth Edition, Text Revision (DSM-IV-TR), perceived stress scale (PSS)	Cross-sectional study	Anxiety, Depression
9	Mingprasert et al. [30]	2014	Thailand	Pharmacy students	N/A	277	ST-5	Cross-sectional study	Depression
10	Rajiah et al. [31]	2014	Malaysia	Pharmacy students	19	225	Westside Test Anxiety Scale (WTAS)	Randomized control trial	Anxiety
11	Abbas et al. [32]	2015	Pakistan	Pharmacy students	18–25	433	N/A	Cross-sectional study	Depression
12	Mojs et al. [33]	2015	Poland	Pharmacy students	21 ± 1.98	229	KADS—the Kutcher Adolescent Depression Scale	Cross-sectional study	Depression
13	Phimarn et al. [34]	2015	Thailand	Pharmacy students	18–20	471	Center for Epidemiological Studies-Depression Scale (CES-D)	Cross-sectional study	Depression
14	Balayssac et al. [35]	2018	French	Pharmacy students	22.0 ± 2.3	2575	The hospital anxiety and depression scale (HADS)	Cross-sectional study	Anxiety, Depression
15	Chuemongkon et al. [36]	2018	Thailand	Pharmacy students	21.39	349	CES-D (Center for Epidemiologic Studies-Depression Scale)	Cross-sectional study	Depression, Suicidal ideation
16	Silva et al. [37]	2018	Portugal	Pharmacy students	21 ± 2.6	410	Hospital Anxiety and Depression Scale (HADS) Perceived Stress Scale (PSS)	Cross-sectional study	Anxiety, Depression
17	Aluh et al. [38]	2019	Nigerian	Pharmacy students	18–30	118	N/A	Cross-sectional study	Stress, Depression
18	Nahas et al. [39]	2019	Malaysia	Pharmacy students	N/A	78	Patient Health Questionaire-9 (PHQ-9)	Cross-sectional study	Depression
19	Fischbein et al. [5]	2019	USA	Pharmacy students	26.7 ± 1.1	159	Nine-item Patient-Health-Questionnaire-9 (PHQ9), Generalized Anxiety Disorder 7-Item (GAD-7)	Cross-sectional study	Depression, Anxiety
20	Hamasha et al. [40]	2019	Saudi Arabia	Pharmacy	N/A	33	Becks Depression Inventory (BDI)	Cross-sectional study	Depression
21	Khaliq et al. [41]	2019	Pakistan	Pharmacy students	N/A	456	Beck’s Depression Inventory (BDI)	Cross-sectional study	Depression
22	Bresolin et al. [42]	2020	Brazil	Pharmacy students	18–22	105	The Beck Depression Inventory version II	Cross-sectional study	Depression
23	Aluh et al. [43]	2020	Nigeria	Pharmacy students	22.57 ± 3.39	408	Depression, Anxiety and Stress Scale (DASS)-21	Cross-sectional study	Depression, Anxiety, Stress
24	Kamil et al. [44]	2020	Sudan	Pharmacy students.	N/A	137	An online questionnaire	Cross-sectional study	Depression
26	Radeef et al. [45]	2020	Malaysia	Pharmacy students	N/A	223	Depression Anxiety, Stress Scale (DASS-21)	Cross-sectional study	Depression, Anxiety, Stress,
27	Samreen et al. [46]	2020	Saudi Arabia	Pharmacy students	18–30	170	The General Anxiety Disorder-7 (GAD-7)	Cross-sectional study	Anxiety
25	Yusof et al. [47]	2020	Malaysia	Pharmacy students	18–29	610	Depression Anxiety Stress Scale-42 (DASS-42)	Cross-sectional study	Depression
28	Ali et al. [48]	2021	Malaysia	Pharmacy students	21.2 ± 1.5	371	Patient Health Questionnaire-9 (PHQ-9)	Cross-sectional study	Depression
29	Campos et al. [49]	2021	Brazil	Pharmacy students	21.7	294	Depression, Anxiety, and Stress Scale (DASS-21)	Cross-sectional study	Depression
30	Khorassani et al. [50]	2021	USA	Pharmacy students	20–23	198	Zung Self-rating Anxiety Scale (SAS)	Cross-sectional study	Anxiety
31	Pate et al. [51]	2021	USA	Pharmacy students	N/A	119	Cognitive Test Anxiety Scale-2 (CTAS-2)	Cross-sectional study	Anxiety
32	Prasomsup et al. [52]	2021	Thailand	Pharmacy students	20.94 ± 1.71	379	Suanprung stress test (SPST-20)	Cross-sectional study	Depression
33	Shangraw et al. [53]	2021	USA	Pharmacy students	18–34	304	Generalized Anxiety Disorder-7 (GAD-7) Patient Health Questionnaire-9 (PHQ-9)	Repeated-measures study	Anxiety, Depression
34	Zakeri et al. [54]	2021	USA	Pharmacy students	N/A	238	Counseling Center Assessment of Psychological Symptoms instrument (CCAPS62)	Cross-sectional study	Anxiety
35	Alamri et al. [55]	2022	Saudi Arabia	Pharmacy students	21.45 ± 1.51	179	Test Anxiety Inventory (TAI)	Cross-sectional study	Anxiety
36	Ansari et al. [56]	2022	Pakistan	Pharmacy students	21.74 ± 2.24	480	State-Trait Anxiety Inventory (STAI)	Cross-sectional study	Anxiety, Suicidal ideation
37	Frajerman et al. [57]	2022	France	Pharmacy student	18–24	233	Hospitalization and Depression scale (HAD)	A cross-sectional study	Anxiety, Depression, Suicidal ideation
38	Hammoudi Halat et al. [58]	2022	Lebanon	Pharmacy students	20 (17–42)	561	Depression Anxiety Stress Scale (DASS-21)	Cross-sectional study	Depression, Anxiety, Stress
39	Mohamed et al. [59]	2022	Egypt	Pharmacy students	22.99 ± 0.84	268	Hamilton Depression Rating Scale (HRS) Patient Health Questionnaire-9 (PHQ-9)	Cross-sectional study	Depression
40	Al Saigh et al. [60]	2022	UAE	Pharmacy students	19.9 ± 2.1	105	Patient Health Questionnaire-9 (PHQ-9)	Cross-sectional study	Depression
41	Sayed et al. [61]	2022	Egypt	Pharmacy students	21.16 ± 1.64 (18–24)	808	Depression Anxiety Stress Scales (DASS-21)	Cross-sectional study	Depression, Anxiety, Stress
42	Tripathi et al. [62]	2022	Saudi Arabia	Pharmacy students	20–24	150	Depression, Anxiety, and Stress Scale (DASS)	Cross-sectional study	Anxiety, Depression, Stress
43	Wahab et al. [63]	2022	Malaysia	Pharmacy students	18–29	610	Depression, Anxiety, and Stress Scale-42 (DASS-42)	Cross-sectional study	Depressive symptoms
44	Annuar et al. [64]	2023	Malaysia	Pharmacy students	22.10 ± 1.50	270	General Anxiety Disorder (GAD-7) Patient Health Questionnaire (PHQ-9)	Cross-sectional study	Anxiety, Depression
45	El-Desoky et al. [65]	2023	USA	Pharmacy students	N/A	214	Counseling Center Assessment of Psychological Symptoms-62 (CCAPS-62)	Cross-sectional study	Anxiety
46	Fadel et al. [66]	2023	Lebanon	Pharmacy students	18–30	866	Depression, Anxiety, and Stress Scale (DASS-21)	Cross-sectional study	Depression, Anxiety, Stress
47	Thi Nhu Hoang et al. [67]	2023	Vietnam	Pharmacy students	21 (19–25)	113	Centre for Epidemiology Studies Depression Scale (CES-D)	Cross-sectional study	Depression
48	Lee et al. [3]	2023	USA	Pharmacy students	24.7 ± 2.81	202	Patient Health Questionnaire-9 (PHQ-9)	Cross-sectional study	Suicidal ideation
49	Nguyen et al. [68]	2023	USA	Pharmacy students	24.7	836	Patient Health Questionnaire-2 (PHQ-2) General Anxiety Disorder-2 (GAD-2)	Cross-sectional study	Depression, Anxiety
50	Akanit et al. [69]	2024	Thailand	Pharmacy students	19–29	313	Suanprung Stress Test-20 (SPST-20) Patient Health Questionnaire-2 (PHQ-2) patient health questionnaire-9 (PHQ-9)	A cross-sectional study	Stress, Depression
51	Sukarnjanaset et al. [70]	2024	Thailand	Pharmacy students	21.89 ± 2.27	179	Patient Health Questionnaire-2 (PHQ-2) Patient Health Questionnaire-9 (PHQ-9)	Cross-sectional study	Depression

**Remark:** N/A, not available.

**Table 2:** Quality assessment results of the included cross-sectional studies based on the NOS.

Author	Number of Stars								
	Selection (Max 5 Stars)				Comparability (Max 2 Stars)		Outcome (Max 3 Stars)		Overall (10)
	1	2	3	4	5	6	7	8	
Sansgiry et al. [23]	★	★	★	★★	★	★	★	★	9
Kanjanasilp et al. [6]	★	★	★	★★	-	★	★	★	8
Alexandrino-Silva et al. [24]	★	★	★	★★	★	★	★	★	9
Khan et al. [25]	★	★	★	★★	★	★	★	★	9
Hunt et al. [27]	★	★	★	★★	★	★	★	-	8
Mingprasert et al. [28]	★	★	★	★★	-	★	★	★	8
Bidwal et al. [29]	★	★	★	★★	★	★	★	★	9
Mingprasert et al. [30]	★	★	★	★★	-	★	★	★	8
Abbas et al. [32]	★	★	★	-	★	★	★	★	7
Mojs et al. [33]	★	★	★	★★	★	★	★	★	9
Phimarn et al. [34]	★	★	★	★★	★	★	★	★	9
Balayssac et al. [35]	★	★	★	★★	★	★	★	★	9
Chuemongkon et al. [36]	★	★	★	★★	★	★	★	★	9
Silva et al. [37]	★	★	★	★★	★	★	★	★	9
Aluh et al. [38]	★	★	★	★★	★	★	★	★	9
Nahas et al. [39]	★	★	★	★★	-	★	★	★	8
Fischbein et al. [5]	★	★	★	★★	★	★	★	★	9
Hamasha et al. [40]	★	★	★	★★	-	★	★	★	8
Khaliq et al. [41]	★	★	★	★★	-	★	★	★	7
Bresolin et al. [42]	★	★	★	★★	★	★	★	★	9
Aluh et al. [43]	★	★	★	★★	★	★	★	★	9
Kamil et al. [44]	★	★	★	★★	-	★	★	★	8
Radeef et al. [45]	★	★	★	★★	★	★	★	★	9
Samreen et al. [46]	★	★	★	★★	★	★	★	★	9
Yusof et al. [47]	★	★	★	★★	★	★	★	★	9
Ali et al. [48]	★	★	★	★★	★	★	★	★	9
Campos et al. [49]	★	★	★	★★	★	★	★	★	9
Khorassani et al. [50]	★	★	★	★★	★	★	★	★	9
Pate et al. [51]	★	★	★	★★	★	★	★	★	9
Prasomsup et al. [52]	★	★	★	★★	★	★	★	★	9
Zakeri et al. [54]	★	★	★	★★	-	★	★	★	8
Alamri et al. [55]	★	★	★	★★	★	★	★	★	9
Ansari et al. [56]	★	★	★	★★	★	★	★	★	9
Frajerman et al. [57]	★	★	★	★★	★	★	★	★	9
Hammoudi Halat et al. [58]	★	★	★	★★	★	★	★	★	9
Mohamed et al. [59]	★	★	★	★★	★	★	★	★	9
Al Saigh et al. [60]	★	★	★	★★	★	★	★	★	9
Sayed et al. [61]	★	★	★	★★	★	★	★	★	9
Tripathi et al. [62]	★	★	★	★★	★	★	★	★	9
Wahab et al. [63]	★	★	★	★★	★	★	★	★	9
Annuar et al. [64]	★	★	★	★★	★	★	★	★	9
El-Desoky et al. [65]	★	★	★	★★	-	★	★	★	8
Fadel et al. [66]	★	★	★	★★	★	★	★	★	9
Thi Nhu Hoang et al. [67]	★	★	★	★★	★	★	★	★	9
Lee et al. [3]	★	★	★	★★	★	★	★	★	9
Nguyen et al. [68]	★	★	★	★★	★	★	★	★	9
Akanit et al. [69]	★	★	★	★★	★	★	★	★	9
Sukarnjanaset et al. [70]	★	★	★	★★	★	★	★	★	9
Gonçalves et al. [26]	★	-	★	★★	★	-	★	-	6
Shangraw et al. [53]	★	★	★	★	★	★	★	-	7

**Remark:** ★ represents a score of 1 point; - denotes a score of 0.

**Table 3:** Risk of bias assessment for each included study.

Study	D1: Bias Arising from the Randomization Process	D2: Bias Due to Deviations from Intended Intervention	D3: Bias Due to Missing Outcome Data	D4: Bias in Measurement of the Outcome	D5: Bias in Selection of Reported Results	Overall
Rajiah et al. [31]	Low	High	Low	Some concern	Low	High

**Figure A1:**
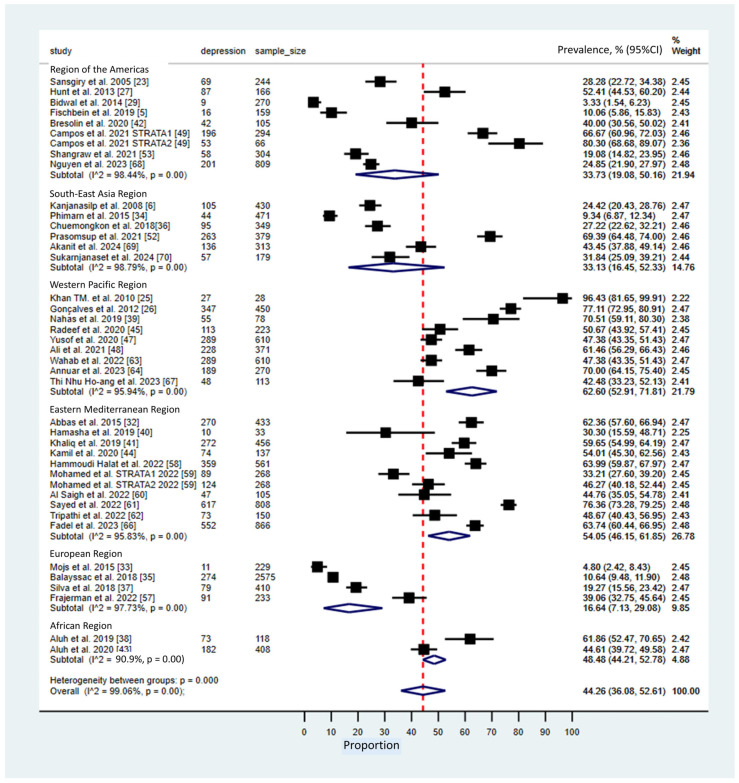
Subgroup analysis prevalence of depression among pharmacy students across WHO regions. (The solid black square (■) represents the point estimate of the observed outcome. The diamond square (◆) denotes the pooled or overall estimate derived from the aggregated data. The dotted line (---) indicates the final pooled estimate).

**Figure A2:**
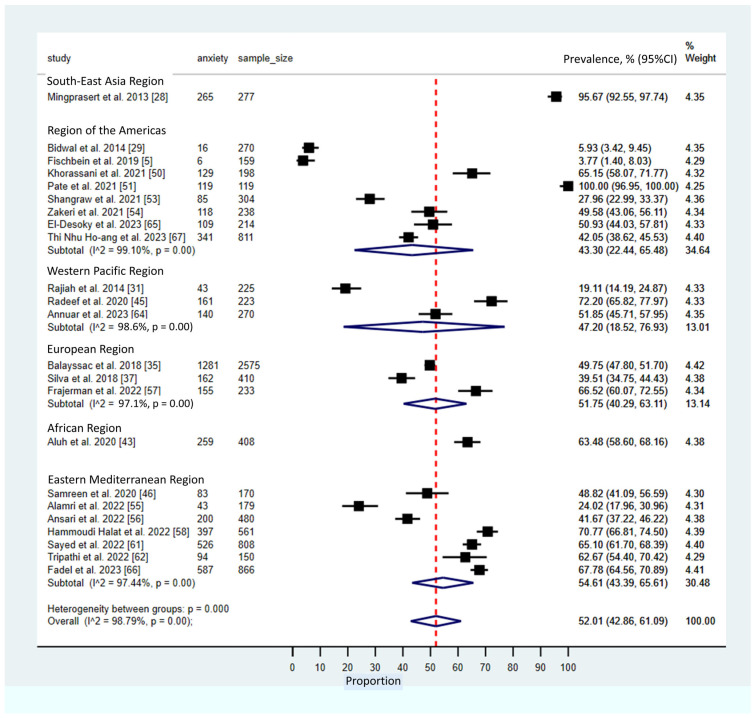
Subgroup analysis prevalence of anxiety among pharmacy students across WHO regions. (The solid black square (■) represents the point estimate of the observed outcome. The diamond square (◆) denotes the pooled or overall estimate derived from the aggregated data. The dotted line (---) indicates the final pooled estimate).

**Figure A3:**
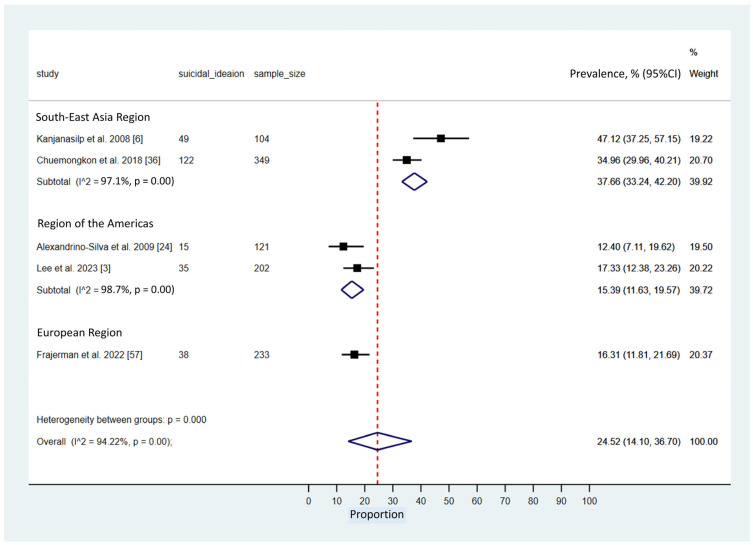
Subgroup analysis prevalence of suicidal ideation among pharmacy students across WHO regions. (The solid black square (■) represents the point estimate of the observed outcome. The diamond square (◆) denotes the pooled or overall estimate derived from the aggregated data. The dotted line (---) indicates the final pooled estimate).

## Data Availability

The datasets used and/or analyzed during the current study are available from the corresponding author on reasonable request.

## Supplementary Materials

The following supporting information can be downloaded at: https://www.mdpi.com/article/10.3390/ijerph23020155/s1, Table S1: PRISMA 2020 checklist [115].

## Abbreviations

The following abbreviations are used in this manuscript:

BDI	Beck Depression Inventory
BSI	Beck Scale for Suicidal Ideation
CCAPS-62	Counseling Center Assessment of Psychological Symptoms-62
CES-D	Center for Epidemiological Studies–Depression Scale
CI	Confidence interval
CTAS-2	Cognitive Test Anxiety Scale–2nd Edition
DASS	Depression Anxiety Stress Scale
DSM	Diagnostic and Statistical Manual of Mental Disorders
GAD-7	Generalized Anxiety Disorder-7
GDP	Gross domestic product
GRADE	The Grading of Recommendations Assessment, Development, and Evaluation
HADS	Hospital Anxiety and Depression Scale; DASS
KADS	Kutcher Adolescent Depression Scale
LMICs	Low- and Middle-Income Countries
NOS	The Newcastle-Ottawa quality assessment scale
PHQ	Patient Health Questionnaire
PIs	Prediction intervals
PRISMA	The Preferred Reporting Items for Systematic Reviews and Meta-Analyses
PSS	Perceived Stress Scale
SAS	Zung Self-Rating Anxiety Scale
SPST-20	Suanprung Stress Test-20
STAI	State-Trait Anxiety Inventory
ST-5	Stress Test Questionnaire
TAI	Test Anxiety Inventory
WHO	World Health Organization
WTAS	Westside Test Anxiety Scale

## Appendix A

### Appendix A.1

**Table A1:** Search strategies.

Database	Step	Search Algorithm	Items Found
PubMed	#1	“Depression” [MeSH]	684,254
	#2	“Depressive illness”	3505
	#3	“Depressive disorder”	134,903
	#4	Anxiety” [MeSH]	367,987
	#5	Stress [MeSH]	119,192
	#6	Suicide	129,659
	#7	“Suicidal ideation”	24,894
	#8	“Pharmacy student” [MeSH]	961
	#9	“Pharmacy school”	3350
	#10	(“Depression” [MeSH] OR “Depressive disorder” OR “Depressive illness”) OR (“Anxiety” [MeSH]) OR (“Stress, Psychological” [MeSH]) OR (Suicide OR “Suicidal ideation”) AND (“Students, Pharmacy” [MeSH] OR “Pharmacy school”)	29
Scopus	#1	Depression	1,001,772
	#2	“Depressive illness”	5003
	#3	“Depressive disorder”	135,154
	#4	Depres*	1,149,283
	#5	Anxiety	620,566
	#6	Stress	338,538
	#7	Suicide	49,585
	#8	“Suicidal ideation”	41,403
	#9	“Pharmacy student”	10,414
	#10	“Pharmacy school”	2461
	#11	(Depression OR “Depressive illness” OR “Depressive disorder” OR Depres* OR Anxiety OR Stress OR Suicide OR “Suicidal ideation”) AND (“Pharmacy student” OR “Pharmacy school”)	299
ScienceDirect	#1	Depression	1,037,892
	#2	“Depressive illness”	16,176
	#3	“Depressive disorder”	112,073
	#4	Anxiety	713,648
	#5	Stress	912,115
	#6	Suicide	188,757
	#7	“Suicidal ideation”	41,723
	#8	“Pharmacy student”	7981
	#9	“Pharmacy school”	11,142
	#10	(Depression OR “Depressive illness” OR “Depressive disorder”) OR (Anxiety) OR (Stress) OR (Suicide OR “Suicidal ideation”) AND (“Pharmacy student” OR “Pharmacy school”)	1360
